# P2-type purinergic signaling in the regulation of pancreatic β-cell functional plasticity as a promising novel therapeutic approach for the treatment of type 2 diabetes?

**DOI:** 10.3389/fendo.2022.1099152

**Published:** 2022-12-09

**Authors:** Nour Mesto, Jamileh Movassat, Cécile Tourrel-Cuzin

**Affiliations:** Université Paris Cité, Unité de Biologie Fonctionnelle et Adaptative, CNRS UMR 8251, Team “Biologie et Pathologie du Pancréas Endocrine”, Paris, France

**Keywords:** P2 purinergic receptors, P2X signaling, P2Y signaling, pancreatic ß-cell function, pancreatic ß-cell plasticity, diabetes

## Abstract

Diabetes Mellitus is a metabolic disorder characterized by a chronic hyperglycemia due to an impaired insulin secretion and a decreased in peripheral insulin sensitivity. This disease is a major public health problem due to it sharp prevalence. Therefore, it is crucial to readapt therapeutic approaches for the treatment of this pathology. One of the strategies would be through P2-type purinergic receptors pathway *via* ATP binding. In addition to its well-known role as an intracellular energy intermediary in numerous biochemical and physiological processes, ATP is also an important extracellular signaling molecule. ATP mediates its effects by binding and activating two classes of P2 purinoreceptors: P2X receptors that are ligand-gated ion channel receptors, existing in seven isoforms (P2X 1 to 7) and P2Y receptors that are G-protein coupled receptors, existing in eight isoforms (P2Y 1/2/4/6/11/12/13/14). These receptors are ubiquitously distributed and involved in numerous physiological processes in several tissues. The concept of purinergic signaling, originally formulated by Geoffrey Burnstock (1929-2020), was also found to mediate various responses in the pancreas. Several studies have shown that P2 receptors are expressed in the endocrine pancreas, notably in β cells, where ATP could modulate their function but also their plasticity and thus play a physiological role in stimulating insulin secretion to face some metabolic demands. In this review, we provide a historical perspective and summarize current knowledge on P2-type purinergic signaling in the regulation of pancreatic β-cell functional plasticity, which would be a promising novel therapeutic approach for the treatment of type 2 diabetes.

## 1 Introduction on purinergic signaling

The intracellular adenosine triphosphate (ATP) was first known for its role as an energy molecule involved in the storage, transport and energy regulation of cellular metabolism. In pancreatic β-cells, intracellular ATP plays a major role in stimulating insulin secretion and maintaining glucose homeostasis. ATP, generated by glycolysis and oxidative phosphorylation, causes the closure of ATP-dependent potassium channels (K^+^-ATP), leading to an accumulation of intracellular potassium (1, 2) and to membrane depolarization. This will induce the opening of voltage-gated calcium channels (VDCC) and an influx of Ca^2+^, resulting in an increase in the concentration of intracellular calcium (3). The important influx of calcium is the main trigger of insulin granule exocytosis (4–8).

In addition to its intracellular role, ATP with ADP and adenosine has also been recognized as an extracellular signaling molecule that can stimulate signaling pathways by binding to purinergic receptors. This concept was originally proposed and formulated by Geoffrey Burnstock in 1972 (9, 10). Extracellular ATP can be released by various systems (exocytosis of secretory granules, vesicular transports and membrane channels such as connexin, pannexin hemi-channels and voltage-gated anion channels) and stimulate purinergic receptors. Nevertheless, ATP can also be released from damaged cells (11). The increase in ATP levels in the extracellular space activates ectonucleotidases (ectonucleoside triphosphate diphosphohydrolases type I or NTPDases) which will rapidly degrade ATP into ADP and adenosine which can also activate some purinergic receptors (12).

Purinergic signaling is the result from the binding of extracellular ATP and its metabolites to purinergic receptors: P1 receptors which have a high affinity for adenosine, a degradation product of ATP by ectonucleotidases and P2 receptors that are activated mainly by ATP, but also by ADP, UTP and UDP and divided into two subgroups P2X and P2Y receptors.

**Figure 1:**
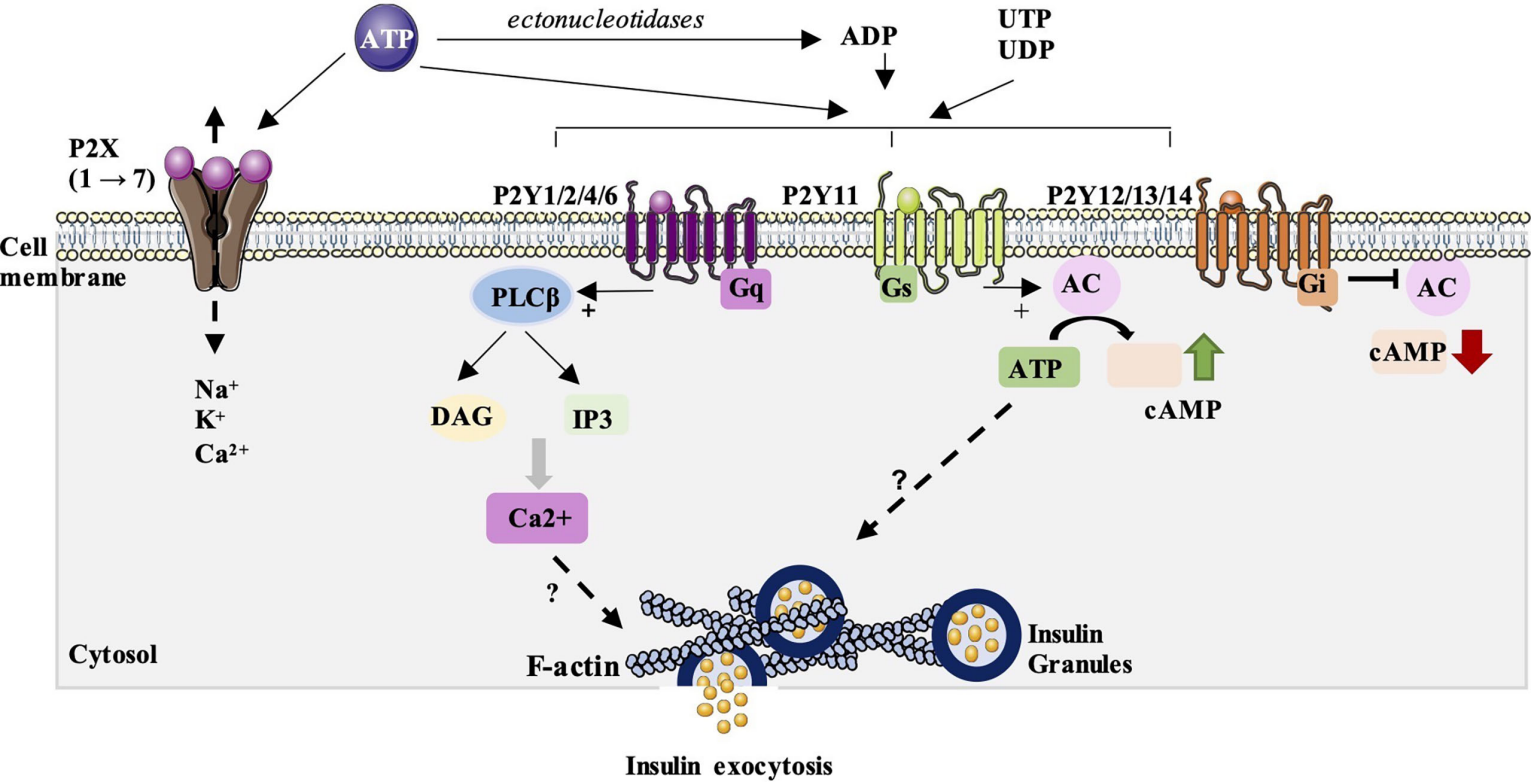
P2 purinergic receptors signaling and their possible involvement in insulin secretion. P2X receptors are ionotropic, ligand-gated ion channel receptors which are permeable to Na^+^, K^+^ and Ca^2+^ ions after activation by ATP. To date, seven subunits (P2X_1→7_) has been identified. Their impact on insulin secretion is quite controversial, with stimulatory and/or inhibitory effects described. P2Y receptors are metabotropic receptors from the large family of G protein-coupled receptors. They can activated by ATP but also by ADP, UTP and UDP. Eight receptor subtypes have been identified and subdivided into two P2Y receptor subfamilies: P2Y1, 2, 4 and 6 which are mainly coupled to G_q_ proteins and activate phospholipase C β pathway (PLCβ). P2Y11 can also be coupled to Gs proteins and thus stimulate adenylate cyclase (AC) and the synthesis of the second messenger, cAMP. The second group includes the P2Y12, 13 and 14 isoforms and are mainly coupled to Gi proteins and inhibit adenylate cyclase and cAMP synthesis. A study done by our team has showed that P2Y activation can stimulate glucose-induced insulin secretion by acting on the distal steps of insulin exocytosis including the subcortical actin network and insulin granules exocytosis. However, the mechanisms involved in this stimulation remains unknown.

P2X receptors are ionotropic, ligand-gated ion channel receptors which are permeable to Na^+^, K^+^ and Ca^2+^ ions after activation by ATP (**Figure 1**). To date, seven subunits (P2X 1→7) has been identified in many cells of various species, including humans (hP2XR), rats (rP2XR), mice (mP2XR) and zebrafish (zP2X4.1R), where they contribute to the control of many physiological functions (13–19). The structural properties and pharmacology on this class of receptors have been well described in a recent review in 2021 (20).

P2Y receptors are metabotropic receptors from the large family of G protein-coupled receptors. To date, eight receptor subtypes have been identified and functionally defined (P2Y1, 2, 4,6, 11, 12, 13, 14) (21–24). These eight subtypes have been subdivided into two P2Y receptor subfamilies based on their associated signaling pathways (23, 25) (**Figure 1**). The first group consists of isoforms P2Y1, 2, 4, 6 and 11. These subtypes are mainly coupled to G_q_/G_11_ proteins which activate phospholipase C (PLC), thus inducing the synthesis of two second messengers: IP3, which mobilizes calcium from the endoplasmic reticulum, and diacylglycerol (DAG) which stimulates the protein kinase C pathway. P2Y11 can also be coupled to Gs proteins and thus stimulate adenylate cyclase and the synthesis of the second messenger, cAMP. The second group includes the P2Y12, 13 and 14 isoforms and are mainly coupled to Gi/o proteins and inhibit adenylate cyclase and cAMP synthesis. The structural properties and pharmacology of these receptors have been well described in the recent review of Jacobson et al. (26).

P2-Purinergic receptors are widely distributed in all tissues and involved in many biological activities such as exocrine and endocrine secretions, platelet aggregation, vascular tone, nociception, neuromodulation but also in cell proliferation, migration, differentiation, inflammation and cell death (16, 27, 28). Their ubiquitous expression in metabolic tissues, notably in the pancreas, have also suggested quite early a role for these receptors in the regulation of the metabolic state and glucose metabolism. In exocrine pancreas, several studies have shown the expression of P2X and P2Y subtypes in acinar and ductal cells in many species (rat, mouse and human) and their roles in modulating cell proliferation and exocrine secretion (29, 30). In α-cells, there is little and controversial information on the role of purinergic signalling in these cells. Some reports have shown that ATP, by activating P2Y receptors, can stimulate glucagon secretion in mouse islets (31) while others have shown an inhibitory effect of P2Y-signaling (32). Thus, the role of purinergic signaling in glucagon secretion needs to be further investigated in order to better understand its involvement in α-cells.

The aim of this review is to gather, at best, the studies carried out to better understand the involvement of P2 purinergic signaling in the endocrine pancreas, most notably in pancreatic β-cells and the possible therapeutic potential that these receptors may present for the treatment of diabetes pathogenesis.

## 2 Extracellular ATP in regulation of pancreatic ß-cell function

### 2.1 Modulation of insulin release by P2R signaling

The first study on the role of purinergic signaling in insulin secretion was first carried out by Rodrigue-Candela et al. in 1963. They have shown that ATP stimulates insulin secretion from β-cells of rabbit pancreas (33). These observations were later confirmed on primate pancreas (34). The stimulatory effect of ATP on insulin secretion was then demonstrated in isolated and perfused rat pancreases (35–37) and on hamster pancreases (38). The use of more potent agonists, in particular, ADP and its analogues (α-β-methylene, ADPβS or ADPγS) have made it possible to show greater stimulation of insulin secretion but which requires stimulating concentrations of glucose (8.3mM) (39–42) while the use of a P2 receptor antagonist abolishes the stimulatory effect of ATP on insulin secretion (37). Stimulation by glucose or by potassium channel inhibitors such as glibenclamide has made it possible to demonstrate the presence of ATP in the secretory granules and in particular in the insulin granules (43–45). ATP was also shown to be co-secreted with insulin during granule exocytosis and amplified glucose-induced insulin secretion by stimulating P2 receptors (46–48) and modulating the biphasic response insulin secretion in a dose-dependent manner (49, 50). Furthermore, the study by Fernandez-Alvarez et al. showed that P2Y receptor agonists (α, β-methylene ATP or ADPβS) amplify insulin secretion from human islets (51).

Interestingly, it has been reported that ATP can be released during the process of “kiss and run” exocytosis without insulin being secreted (48) suggesting that ATP can act as an autocrine regulator of insulin secretion. ATP can also be released from nerve endings and play a role in the neuronal control of insulin secretion (52). Studies have shown that ATP can be stored and co-secreted with acetylcholine and act synergistically on insulin secretion, particularly in the cephalic phase (pre-prandial period) (53). It was shown that ATP, by binding to P2X or P2Y receptors activates various intracellular pathways, including the K^+^/ATP-dependent pathway or the Ca^2+^ pathway. Consequently, it increases β-cell insulin secretion from many models including human β-cells (54, 55).

At the opposite, it has been shown that ATP can also exert an inhibitory effect on insulin secretion. Contrary to what has been observed on the stimulatory effect of ATP in rats, a decrease in insulin secretion in response to ATP has been shown in mice (39). This inhibition was later confirmed by two studies which showed, using membrane capacitance and calcium flux measurement techniques, on MIN6 β-cells, that ATP reduces by 60% the exocytosis induced by depolarization, by interfering with the exocytosis machinery (56). The ATP inhibitory effect observed in mice does not seem to be due to the differences in the expression profile of these receptors between the different species since a study reported that the use of P2 receptor antagonist amplifies the insulin secretion of rat islets in response to stimulating concentrations of glucose (57).

### 2.2 P2X signaling in pancreatic β-cells

Using various approaches, numerous studies, including ours, have identified the presence of the seven subtypes of P2X receptors (1 to 7) in rat and mouse islet β-cells and the INS1 β-cell line but their role remains so contradictory (42, 58, 59). Electrophysiological, RT-PCR and immunocytochemical studies showed that P2X1, P2X3 and P2X4 are expressed in adult rats and mice β-cells (60–62) and that P2X1 and P2X3 can be rapidly desensitized, suggesting that the paracrine or neuronal activation of these receptors would contribute to maintain insulin secretion induced by high-glucose or acetylcholine (62). The expression of P2X3 and P2X5 has also been reported in human β-cells and it was shown that P2X3 isoform could have an amplifying effect on insulin secretion (55). However, in the rat β-cell line, P2X3 receptors appear to negatively regulate insulin secretion independently of extracellular glucose concentrations (59). As for the P2X7 receptors, several studies have shown that P2X7 receptors are expressed in mouse islets and β-cell lines (MIN6). These same studies have reported that P2X7 is downregulated in β-cells from type 2 diabetic patients but highly expressed in obese patients and may be involved in the secretion of pro-inflammatory cytokines such as interleukin-1β (63, 64). In INS-1E β-cell line, the activation of P2X7 receptors regulates ATP secretion and intracellular calcium signaling and consequently regulates insulin secretion (29). However, it was reported that activation of P2X7 inhibits insulin secretion in response to glucose (65). Studies carried out on knockout mice for the P2X7 receptor showed the impact of these receptors on glucose homeostasis (63). P2X7^-/-^ mice exhibit hyperglycemia, glucose intolerance, and impaired β-cell function when fed a high-fat diet and are unable to increase β-cell mass in response to increased nutrient load. The islets from these mice also show increased β-cell apoptosis (63). Several studies have also linked the P2X7 isoform to the pathogenesis of type 1 diabetes (T1D). Indeed, knockout of the P2X7 receptor prevents streptozotocin-induced T1D in mice (66) and inhibition of this receptor by oxidized ATP delays islet graft rejection (67).

### 2.3 P2Y signaling in pancreatic β-cells

Numerous studies have reported the impact of P2Y receptors in modulating insulin secretion in the pancreatic β-cells. Bertrand et al. showed for the first time that P2Y receptors can modulate the biphasic response of insulin secretion in rat pancreas (49). This same team later reported that ADPβS, a preferential agonist of P2Y1 receptors, activates insulin secretion from perfused pancreas and isolated rat islets (41, 50, 68). Fischer et al. also showed that the use of a potent P2Y1 agonist significantly amplifies the insulin secretion in response to glucose from isolated islets and perfused rat pancreases (69). More recently, the activation of P2Ys, with more specific agonists than previously used, including 2Me(S)ATP-α-β, has been shown to potentiate insulin secretion in response to glucose from rat islets (70). A study by Léon et al. on P2Y1^-/-^ mice shows that these mice develop hyperglycemia as well as a tendency towards glucose intolerance and increased insulin secretion compared to WT mice. These results indicate that P2Y1 receptor play an important role in maintaining glucose homeostasis (71). Nevertheless, the study by Ohtani et al. shows that activation of P2Y1 and P2Y6 decreases glucose-induced insulin secretion from mouse islets (72). Later, this same team showed that extracellular ATP increased insulin secretion from mouse islets, but that exposure to high concentrations of ATP counteracted this increase, probably *via* the P2Y1 and P2X4 receptors (73).

We and others have subsequently shown, using RT-PCR and western blot approaches, that the β-cell line derived from rat insulinoma, INS-1, expresses the six isoforms of P2Y (P2Y1, 2, 4, 6, 12 and 13) (59, 74, 75). P2Y1, P2Y6 and P2Y13 are also expressed in mouse β-TC6 cells and in mouse β cells and islets (31, 72). In humans, the P2Y2, 4, 11 and 12 isoforms have been cloned and characterized in the human pancreas (76, 77). In fact, our study carried out in type 2 diabetic rat model, the Goto-Kakizaki rat and in Wistar healthy rats showed that the level of expression of P2Y receptors varies significantly according to glucose environment. We found that P2Y1 was significantly downregulated compared to Wistar rats but the level of expression of P2Y4 was not modified. However, the level of expression of P2Y12 and P2Y13 was significantly increased in Goto-Kakizaki diabetic islets (75). Interestingly, in that same study, we showed that the activation of P2Y receptors in rat β-cell line, INS-1 832/13 β-cells, with impaired insulin secretion following exposure to elevated glucose levels, restores glucose-stimulated insulin secretion (GSIS) competence through the distal steps of insulin exocytosis, by increasing insulin granules exocytosis and the reorganization of the subcortical actin network (**Figure 1**). The activation of P2Y receptors with 2Me(S)ATP also amplified GSIS of healthy Wistar rat islets and, more interesting, partially restored the altered GSIS of diabetic islets from the type 2 diabetic Goto-Kakizaki (GK) rats. Our results emphasize the beneficial effects of the activation of P2Y receptors in insulin secretion on pancreatic β-cells especially in a diabetic context (75). In addition, we showed that the injection of glucose with 2Me(S)ATP decreases significantly the blood glucose levels in the type 2 diabetic GK rats compared to untreated GK rats and this by increasing the glucose-induced insulin secretion and blood glucose clearance by peripheral tissues. Thus, our results show that acute injection of 2Me(S)ATP restores partially the diabetic state of this diabetic rats (75). Our study, along with those cited above, suggests that P2-purinergic signaling may be a good candidate for the treatment of metabolic diseases such as obesity and diabetes.

## 3 P2 receptors in regulation of ß-cell survival

**Figure 2:**
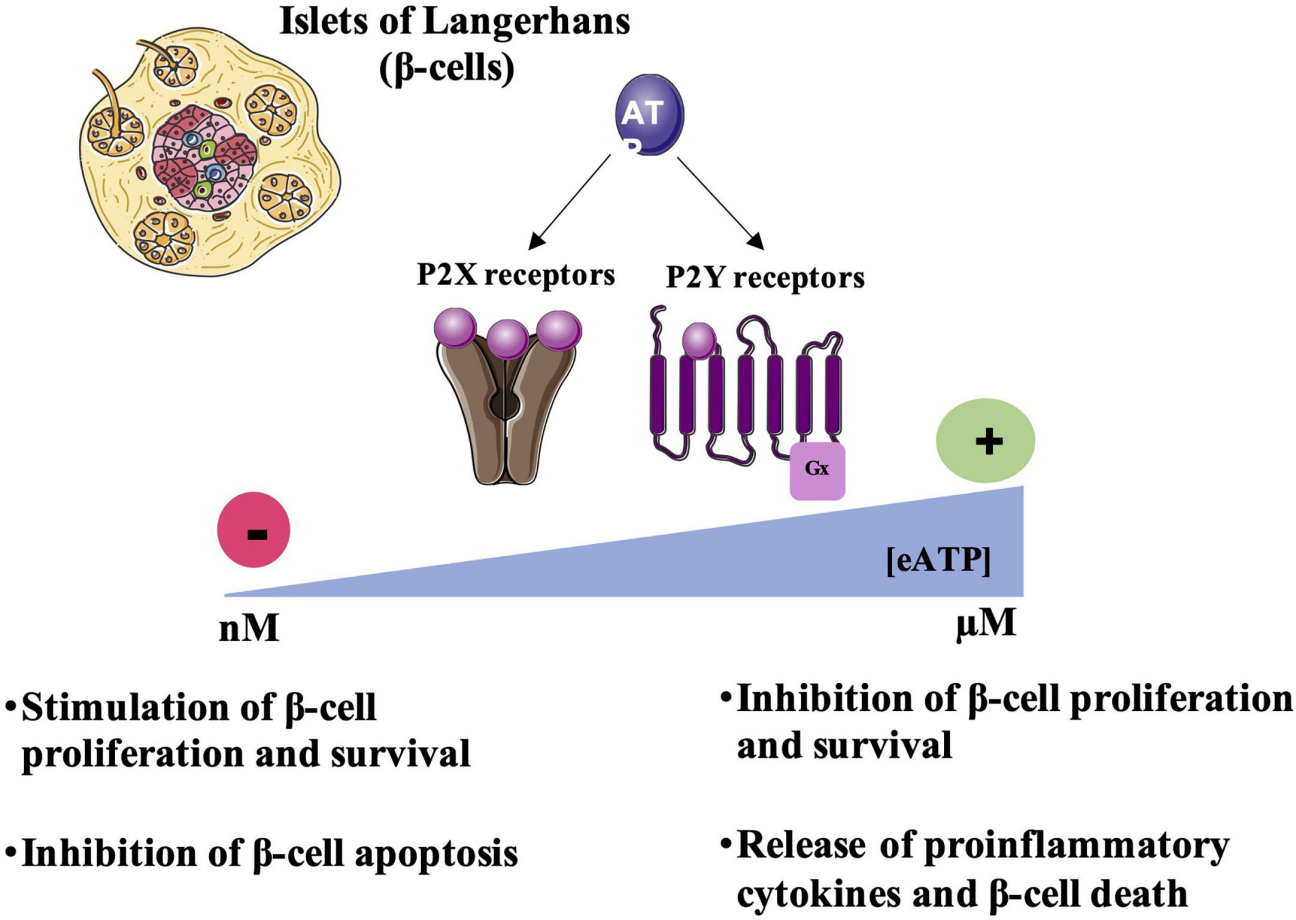
Regulation of β-cell survival by P2R signaling. Under physiological conditions, extracellular ATP concentration [eATP] is in the nanomolar range. Thus, eATP by activating P2 receptors can stimulate β-cell proliferation and survival and inhibit the release of proinflammatory cytokines preventing β-cell death. However, at the inflammation sites or tissue damage, eATP concentrations increases and reaches micromolar ranges activating P2 receptors thus inhibiting β-cell proliferation and inducing the release of proinflammatory cytokines leading to β-cell death.

In addition to all these studies showing the important role of purinergic P2 signaling on the insulin secretory function of β-cells, the presence of numerous isoforms of P2 receptors in pancreatic islets has also raised interest in their role in other processes such as their possible involvement in inflammatory, proliferative and apoptotic pathways (**Figure 2**).

Numerous studies have shown that ATP and its analogues could be involved in tissue remodeling, in particular in the regulation and regeneration of tissues such as the nervous system or the liver (78). A study in the brain have shown that the stimulation of purinergic receptors induces the proliferation of astrocytes indicating the role of these receptors in the remodeling and in the proliferation of neuronal cells (79, 80). Another study carried out on P2X4^-/-^ mice showed that the P2X4 receptor contributes to hepatic regeneration after hepatectomy, suggesting the role of this isoform in the protection and proliferation of hepatocytes (81). In the pancreas, it was shown that the activation of P2Y6 isoform protects β-cells from cell death induced by the pro-inflammatory cytokine, TNF-α (Tumor Necrosis Factor-α) while the activation of P2Y13 in mouse β-cell line, MIN6C4, stimulates caspase-3 activity and reduces cell proliferation (82). These effects were abolished using P2Y13 antagonist, MRS2211 (82). ATP-induced apoptosis was also shown in the HIT-T15 hamster β cell line (83). Moreover, in β-TC6 cells, proliferation was inhibited by micromolar concentrations of ATP, probably *via* the activation of P2X4 receptor. A reduction in the viability of these cells has also been noted, but the results do not support the involvement of a specific P2 receptor subtype (73). Moreover, hyperglycemia in type 2 diabetes pathogenesis promotes extracellular ATP release activating P2X7 receptors and leading to the release of proinflammatory cytokines and β-cell apoptosis (19).

The low-grade chronic inflammation observed in type 2 diabetic patients constitutes a stimulus inducing migration and infiltration of macrophages and immune cells in many tissues including the endocrine pancreas (84, 85). The consequence of this inflammation is an increased dysfunction of the islets of Langerhans which aggravates the decrease in insulin secretion and induces apoptosis of the β cells thus reducing the β-cell mass. Therefore, the reconstitution of a new pool of functional β-cells constitutes a promising therapeutic approach for the treatment of this pathology. Thus, based on the data cited previously, it would be interesting to deepen our knowledge and study the impact of P2-purinergic receptors in the induction of inflammatory and pro-apoptotic processes as well as their potential trophic effect in pancreatic β-cells, notably in diabetic models.

## 4 Conclusions

Since the first cloning of the P2X and P2Y receptors in the 1990s, many subtypes have been identified and their roles in large systems, tissues and cells are better characterized. The therapeutic hopes raised in the field of diabetic pathologies are an encouragement to continue efforts in this direction and to extend the field of investigation to the field of functional plasticity of pancreatic β-cells in which ATP and its purinergic receptors seem to have a physiological and/or physiopathological action.

## Author contributions

NM wrote the manuscript and produced the figures. JM reviewed the manuscript. CT-C designed and critically reviewed the manuscript. All authors contributed to the article and approved the submitted version.
